# Patient Satisfaction With Healthcare Service Quality and Its Associated Factors at One Polyclinic in Hanoi, Vietnam

**DOI:** 10.3389/ijph.2022.1605055

**Published:** 2022-11-10

**Authors:** Nguyen Duc Thanh, Bui Thi My Anh, Chu Huyen Xiem, Pham Quynh Anh, Pham Hung Tien, Nguyen Thi Phuong Thanh, Cao Huu Quang, Vu Thu Ha, Phung Thanh Hung

**Affiliations:** ^1^ Department of Hospital Management, Health Management Training Institute, Hanoi University of Public Health, Hanoi, Vietnam; ^2^ Department of Health Organization and Management, School of Preventive Medicine and Public Health, Hanoi Medical University, Hanoi, Vietnam; ^3^ Department of Health Organization and Management, Health Management Training Institute, Hanoi University of Public Health, Hanoi, Vietnam; ^4^ Faculty of Clinical Medicine, Hanoi University of Public Health, Hanoi, Vietnam; ^5^ Center for Quality Assurance and Examination, Hanoi University of Public Health, Hanoi, Vietnam; ^6^ Department of Student Affairs, Hanoi University of Public Health, Hanoi, Vietnam

**Keywords:** health service quality, reliability, validity, outpatient satisfaction, public polyclinic, Vietnam

## Abstract

**Introduction**: Patient satisfaction is one of the most important components of measuring healthcare quality.

**Objectives**: The study aimed to evaluate the validity and reliability of the patient satisfaction scale with the quality of health services and its associated factors.

**Methods:** A cross-sectional study was conducted to collect data on patient satisfaction with 301 outpatients at one polyclinic in Hanoi, Vietnam.

**Results:** The overall outpatient satisfaction was 53.5%. There were five factors (facilities, services provision results, information transparency and administrative procedures, accessibility, and interaction and communication of staff) including one major factor with high Eigenvalues coefficient, 22.5 for satisfaction with facility, and four others with lower Eigenvalues coefficient, 3.2, 2.0, 1.5, and 1.2 for satisfaction with service provision results, information transparency and administrative procedures, accessibility, and interaction and communication of staff respectively. All satisfaction-factors show internal consistency reliability, with a Cronbach’s Alpha of over 0.9. The insured are 3.5 times (95% CI: 1.9–6.2) more likely to be satisfied with health services than the uninsured.

**Conclusion:** The patient satisfaction measurement tool should be used for intervention to improve the quality of health services at the clinic.

## Introduction

Patient satisfaction assessment has been widely deployed around the world [1–3]. Patient satisfaction is “when medical services meet the patient’s expectations during treatment” [4]. Patient satisfaction with the quality of the health service they receive is very important, reflecting the quality of the health facility, thereby proposing solutions to improve the quality of the hospital. Providing healthcare services to satisfy customers/patients is a key factor affecting the existence and development of health facilities [5].

In Vietnam, there has been some very interesting content on the topic of satisfaction since 2013, when the Ministry of Health issued guidelines on the medical examination and treatment process as well as Decision No. 4448 approving the project to determine a method of measuring patient satisfaction with public health services [4]. Quality of medical examination and treatment is an important contributor to improving patient satisfaction, and some studies have shown that meeting the needs/expectations of customers/patients will help the hospital achieve the desired “quality of service” [6–8]. Customers will be more likely to come back to health facilities to access their healthcare services once they are satisfied with healthcare service quality.

Since then, many hospitals and health clinics have conducted studies on patient satisfaction and service quality in Vietnam [9–11]. The tool used to measure patient satisfaction with health service quality in these studies is the SERVQUAL tool recommended by the Vietnam Ministry of Health [12–14]. There is an increasing number of Vietnamese health facilities using the tool suggested by the Vietnam Ministry of Health. This tool helps to compare health care quality among health facilities by assessing five components: facilities, services provision results, information transparency and administrative procedures, accessibility, and interaction and communication of staff. However, some aspects of this tool are still general and unclear, which may lead to inaccurate assessment results. It is important to revise and adapt the tool appropriately to the local language to ensure the reliability and validity of the tool.

This study was conducted at one polyclinic in Hanoi, Vietnam. This polyclinic was licensed to operate under License No. 341/BYT-GPHD issued by the Ministry of Health on 23 June 2017 for the purpose of medical examination, treatment, and prevention. The polyclinic is a public healthcare facility that provides medical examination and treatment as well as primary healthcare services with health insurance equivalent to the district level in Vietnam’s healthcare system. Since 2017, there have been no studies on patient satisfaction at this polyclinic. Therefore, a study on patient satisfaction is necessary to provide evidence to improve the quality of health services. In this study, we adapted the satisfaction assessment tool of the Ministry of Health to fit the context of the polyclinic with the following objectives: 1) to validate the patient satisfaction instrument and 2) to identify the factors associated with satisfaction at one polyclinic in Hanoi, Vietnam.

## Methods

### Study Design

A cross-sectional study was used to collect data on patient satisfaction with health services at one polyclinic in Hanoi, Vietnam.

### Study Subjects and Sampling

The sample size for this study was 301 outpatients who came to get preventive and curative day-time care at the clinic. Convenient sampling was applied in this study with about 10 patients being chosen for interview each day.

### Study Instruments

In Vietnam, the Ministry of Health issued the hospital quality criteria for measuring patient satisfaction. A satisfaction-measuring questionnaire was developed with 39 questions. However, almost all of the questions were too vague to answer. Thus, an instrument’s face validity was confirmed through a workshop that was organized to reach a consensus on the patient satisfaction scale that is appropriate for the polyclinic. The workshop’s participants were stakeholders who are university lecturers of hospital management (five participants) and staff of the clinic’s quality assurance department (three participants) and the director board of the clinic (one participant). A voting technique was used to reach the participants’ agreement on the patient satisfaction items and scale.

### Data Collection

The self-reported structured questionnaire was then developed and undertaken with reference to the questionnaire of patient satisfaction developed by the Ministry of Health as mentioned above. The instructors, who were staff members at the Health Management Training Institute, Hanoi University of Public Health with good research skills, conducted data collection sessions.

### Measurement and Variables

The dependent variable was patient satisfaction with health services. The Likert scale with five levels was applied [1]: strongly unsatisfied to [5] strongly satisfied. By summing up the response of 39 questions, the scores more than or equal to the mean score were categorized as satisfied and those less than the mean were categorized as unsatisfied. So, the mean score was used as the cut-off value [15]. The independent variables include socio-demographic characteristics such as age, gender, education, occupation, economic status, and health insurance status.

### Data Management and Analysis

Data were coded, cleaned, and entered into the computer using Epi-data software and analyzed by SPSS 18.0. The instrument’s Convergent validity was identified by exploratory factor analysis. Several criteria were assessed before factor analysis could be done. The correlation co-efficient among items must be over 0.4. Kaiser-Meyer-Oklin must be over the recommended parameter of 0.6 (Kaiser, 1970, 1974) to mean that the sample size was large enough to conduct factor analysis [16, 17]. Test Barlett Sphericity (Barlett, 1954) was statistically meaningful with *p* < 0.05 [18]. Varimax rotation was applied to interpret the identified factors. Factors with Eigen values over 1 should be retained in the analysis [17]. The tool’s internal consistency reliability was confirmed by Cronbach’s Alpha. This value of greater than 0.7 was considered acceptable [19]. Multiple logistic regression analysis was used to predict the factors associated with patient satisfaction with healthcare service quality. Odd ratios and a 95% confidence interval were used to describe the association among the variables. Statistical significance was set at a *p*-value < 0.05.

### Ethical Clearance

The study was approved by the Ethical Review Committee of Hanoi University of Public Health (Decision No 123/2022/YTCC-HD3). All the answers and information of the participants were kept confidential and used for the study purpose only. The studied individuals signed the informed consent form.

## Results

In the sample of 301 participants, male and female accounted for 69.4% and 30.6% respectively. Education was re-coded to form two groups: under college, and college and higher. The rate of those falling into the category of under college was similar to those in the college and higher group. A majority of the sample reported being employed (70.1%). Those who were found to be unemployed accounted for 8.0% and students and retired accounted for 21.9%.

The participants aged between 30 and 39 accounted for the lowest proportion, 25.9%. The highest rate of age group was found in the group from 40 and above, 43.9%. And those who were younger than 30 accounted for 30.2%. Most of the participants had a fair economic status, at 95.7%. Those who used health insurance cards for their healthcare services were three times more prevalent than those who did not use them (Table 1).

**TABLE 1:** General characteristics of the participants who used healthcare services at a polyclinic (Hanoi, Vietnam, 2022).

Characteristics	Number (*n* = 301)	Percent (%)
Age		
Younger than 30	91	30.2
30 to 39	78	25.9
40 and older	132	43.9
Gender		
Female	92	30.6
Male	209	69.4
Education		
Under college	149	49.5
College and higher	152	50.5
Occupation		
Unemployed	24	8.0
Employed	211	70.1
Students, retired	66	21.9
Economic status		
Rich	8	2.6
Fair	288	95.7
Near-poor and Poor	5	1.7
Insurance status		
No	75	24.9
Yes	226	75.1

### Description of Patient Satisfaction Items

The 301 patients who accessed the polyclinic in Hanoi were asked to answer 39 questions about health service satisfaction with a 100% rate of responses. The total score of patient satisfaction was 117–190. This continuous variable was recoded into a dichotomous variable for analysis, the mean score of 171 was used as a cut-off value as described in the method (*Measurement and Variables*). The overall outpatient satisfaction was 53.5%.

The subtotal score of each factor was calculated by summing all the factor’s items. The subtotal mean score of the factor “Accessibility” of 6 items was 26.9 (Min = 18.0; Max = 30.0; SD = 3.14), of the factor “Information transparency and administrative procedures” of 7 items was 30.9 (Min = 21.0; Max = 35.0; SD = 3.81), of the factor “Facilities” of 10 items was 46.1 (Min = 20.0; Max = 50.0; SD = 5.0), of the factor “Interaction and communication of staff” of 6 items was 27.3 (Min = 18.0; Max = 30.0; SD = 3.1), and of the factor “Service supply results” of 10 items was 44.3 (Min = 10.0; Max = 50.0; SD = 5.4). The total score of the patient satisfaction scale was calculated by summing 39 items and the result was from 117 to 190. The higher the score, the more satisfaction is indicated. The mean score of the scale was 171 (Min = 117.0; Max = 190.0; SD = 17.2) (Table 2).

**TABLE 2:** Description of patient satisfaction items at a polyclinic (Hanoi, Vietnam, 2022).

Items (*n* = 301)	Min	Max	Mean	SD
Accessibility				
Signposts help you find the clinic easily	1	5	4.48	0.61
The diagram of the lobby is clear	3	5	4.48	0.60
Easy-to-understand instructions from staff to specialized rooms	2	5	4.53	0.59
Notice of clinic on time of medical examination and treatment is clear	2	5	4.50	0.58
Notice about the time to receive specific subclinical results (tests, X-rays, ultrasound ...) is clear	1	5	4.47	0.65
When you need assistance, it is always met	3	5	4.49	0.58
Total score	18	30	26.9	3.1
Total score as abnormal distribution (Shapiro-Wilk test: *p* < 0.05)	18	30	Median: 28.0	IQRs: 6.0
**Information transparency and administrative procedures**				
The process of medical examination and treatment is publicly notified	1	5	4.50	0.59
You are clearly explained about your illness	3	5	4.53	0.55
You are clearly explained about the treatment	2	5	4.50	0.57
You are clearly explained about the treatment time and the disease progress	3	5	4.50	0.55
You are was consulted and explained clearly about the need for tests, subclinical (tests, ultrasound, X-ray, gastrointestinal endoscopy, ...)	3	5	4.44	0.62
You are consulted and explained clearly about service prices before performing subclinical tests (tests, ultrasound, X-ray, gastrointestinal endoscopy, ...)	1	5	4.37	0.72
Prices for medical services are posted in an easy-to-see position	1	5	4.08	0.96
Total score	21	35	30.9	3.8
Total score as abnormal distribution (Shapiro-Wilk test: *p* < 0.05)	21	35	Median: 31	IQRs: 7
**Facilities**				
The walkway in the clinic is not slippery, does not stagnant water	2	5	4.63	0.55
Arranging full seats waiting for customers	2	5	4.65	0.52
The area in the clinic is spacious, clean, with appropriate temperature control equipment (fans, air conditioners, ...)	2	5	4.65	0.53
Specialized clinic rooms are provided with clean pillows	2	5	4.59	0.58
Toilets are clean with available toilet paper, soap and water	1	5	4.59	0.61
You are provided with hot/cold drinking water	2	5	4.57	0.60
Clinic ensuring privacy for you when conducting medical examination and treatment (blinds, partitions)	1	5	4.51	0.67
Environment, view of the clinic are green	2	5	4.62	0.54
Environment, view of the clinic are clean	2	5	4.65	0.51
Environment, view of the clinic are nice	2	5	4.64	0.53
Total score	20	50	46.1	5.0
Total score as abnormal distribution (Shapiro-Wilk test: *p* < 0.05)	20	50	Median: 50	IQRs: 10
**Interaction and communication of staff**				
Staff always have words, attitude, proper communication, warm and friendly.	2	5	4.49	0.65
You are respected, treated fairly and cared for by the staff	3	5	4.55	0.56
The staff handle the job competently, responding promptly to your needs	3	5	4.54	0.56
You are advised to use medicine, diet, exercise regime and preventive medicine.	3	5	4.55	0.57
The staff does not suggest, ask for gifts and presents, making it difficult for customers	3	5	4.60	0.54
The clothes of the staff are neat, clean and beautiful, not crumpled, wearing a full name plate	3	5	4.60	0.61
Total score	18	30	27.3	3.0
Total score as abnormal distribution (Shapiro-Wilk test: *p* < 0.05)	18	30	Median: 29.0	IQRs: 6.0
**Service supply results**				
You are satisfied with the waiting time for medical examination and treatment	1	5	4.47	0.59
You are satisfied with the time of medical examination and treatment	1	5	4.49	0.63
You are satisfied with the waiting time to perform near-clinical services (tests, X-rays, ultrasound ...)	1	5	4.43	0.67
You are satisfied with the waiting time for receiving the results of subclinical tests (tests, ultrasound, imaging, screening, functional probes, ...)	1	5	4.41	0.60
You are provided with adequate medication instructions	1	5	4.46	0.60
Medical equipment and supplies of the clinic are sufficient to meet your needs	1	5	4.49	0.70
You are satisfied with expected treatment result	1	5	4.34	0.60
You will return to the clinic or introduces relatives/acquaintances (when needed)	1	5	4.34	0.56
Reasonable price for medical examination and treatment services, laboratory tests	1	5	4.48	0.66
The cost of medical examination and treatment is appropriate to your economic condition	1	5	4.38	0.64
Total score	10	50	44.3	5.4
Total score as abnormal distribution (Shapiro-Wilk test: *p* < 0.05)	10	50	Median: 45.0	IQRs:b10.0
Total score of the scale	117	190	171	17.2
Total score as abnormal distribution	117	190	Median: 176	IQRs: 37

### Instrument Validation

Factor analysis was used to identify how many factors there were to explain the patients’ satisfaction with health services at the clinic. Several criteria were assessed before factor analysis could be done. Through the correlation matrix, we found that there were many correlations co-efficient over 0.3. This meant that the items were inter-correlated.

Kaiser-Meyer-Oklin was 0.95, over the recommended parameter of 0.6. This meant that the sample size was large enough to conduct factor analysis. Test Barlett Sphericity was statistically meaningful (*p* < 0.01). Factor analysis (PCA) showed that there were five factors with Eigen values over one, explaining 57.6%, 8.1%, 5.1%, 3.9%, and 3.1% of the variance. In order to interpret these five factors, we applied Varimax rotation. The results indicated that there were five factors with total of 39 items with strong loadings. Factor one “Facility” had 10 items. Factor two “Service provision results” had 10 items. Factor three “Information transparency and administrative procedures” had 07 items. Factor four “Accessibility” had 06 items. And factor five “Interaction and communication of staff” had 06 items. These five factors explained 77.7% of the variance in which factor one contributed 57.6%, factor two contributed 8.1%, factor three contributed 5.1%, factor four contributed 3.9%, and factor five contributed 3.1%. Thus, through Eigenvalues of factor number in the patient satisfaction scale, we identified five main factors with Eigenvalues of 22.5, 3.2, 2.0, 1.5, and 1.2 respectively.

The internal consistency reliability of the satisfaction scale was evaluated by Cronbach’s Alpha. All factors had good reliability with Cronbach’s Alpha over 0.9 (Table 3).

**TABLE 3:** 05 factors measuring patient satisfaction with health services at a polyclinic (Hanoi, Vietnam, 2022).

Items	Facilities (F1)	Service provision results (F2)	Information transparency and administrative procedures (F3)	Accessibility (F4)	Interaction and communication of staff (F5)
	Cronbach’s Alpha = 0.97	Cronbach’s Alpha = 0.96	Cronbach’s Alpha = 0.92	Cronbach’s Alpha = 0.94	Cronbach’s Alpha = 0.95
The walkway in the clinic is not slippery, does not stagnant water	0.80				
Arranging full seats waiting for customers	0.83				
The area in the clinic is spacious, clean, with appropriate temperature control equipment (fans, air conditioners, ...)	0.82				
Specialized clinic rooms are provided with clean pillows	0.81				
Toilets are clean with available toilet paper, soap and water	0.83				
You are provided with hot/cold drinking water	0.81				
Clinic ensuring privacy for you when conducting medical examination and treatment (blinds, partitions)	0.67				
Environment, view of the clinic are green	0.80				
Environment, view of the clinic are clean	0.82				
Environment, view of the clinic are nice	0.82				
You are satisfied with the waiting time for medical examination and treatment		0.72			
You are satisfied with the time of medical examination and treatment		0.74			
You are satisfied with the waiting time to perform near-clinical services (tests, X-rays, ultrasound ...)		0.77			
You are satisfied with the waiting time for receiving the results of subclinical tests (tests, ultrasound, imaging, screening, functional probes, ...)		0.75			
You are provided with adequate medication instructions		0.76			
Medical equipment and supplies of the clinic are sufficient to meet your needs		0.71			
You are satisfied with expected treatment result		0.75			
You will return to the clinic or introduces relatives/acquaintances (when needed)		0.75			
Reasonable price for medical examination and treatment services, laboratory tests		0.73			
The cost of medical examination and treatment is appropriate to your economic condition		0.74			
The process of medical examination and treatment is publicly notified			0.56		
You are clearly explained about your illness			0.70		
You are clearly explained about the treatment			0.78		
You are clearly explained about the treatment time and the disease progress			0.76		
You are was consulted and explained clearly about the need for tests, subclinical (tests, ultrasound, X-ray, gastrointestinal endoscopy, ...)			0.72		
You are consulted and explained clearly about service prices before performing subclinical tests (tests, ultrasound, X-ray, gastrointestinal endoscopy, ...)			0.61		
Prices for medical services are posted in an easy-to-see position			0.40		
Signposts help you find the clinic easily				0.76	
The diagram of the lobby is clear				0.77	
Easy-to-understand instructions from staff to specialized rooms				0.62	
Notice of clinic on time of medical examination and treatment is clear				0.74	
Notice about the time to receive specific subclinical results (tests, X-rays, ultrasound ...) is clear				0.65	
When you need assistance, it is always met				0.67	
Staff always have words, attitude, proper communication, warm and friendly.					0.79
You are respected, treated fairly and cared for by the staff					0.74
The staff handle the job competently, responding promptly to your needs					0.70
You are advised to use medicine, diet, exercise regime and preventive medicine.					0.57
The staff does not suggest, ask for gifts and presents, making it difficult for customers					0.56
The clothes of the staff are neat, clean and beautiful, not crumpled, wearing a full name plate					0.62

*The above table only shows the items with loadings over 0.4.

### Patients’ Satisfaction With Health Services at the Clinic and Associated Factors

Six independent variables such as age, sex, education, occupation, economic status, and insurance status were put into the model for logistic regression analysis with the dependent variable of “patients’ satisfaction” with the mean cut-off. The analysis results showed that only insurance status had a significant association with the dependent variable. The insured were 3.5 times more likely to be satisfied with health service than the uninsured (OR = 3.5, 95% CI = 1.9–6.2). Other variables had no significant association with the dependent variable (Table 4).

**TABLE 4:** Adjusted odds ratio and 95% confidence intervals for measures of patient satisfaction at a polyclinic (Hanoi, Vietnam, 2022).

Variable	Patient satisfaction
	Yes (*n* = 161) vs. No (*n* = 140)
Age	
Younger than 30	—
30 to 39	0.8^a^ (0.4–1.6)^b^
40 and older	0.7 (0.4–1.5)
Gender	
Male	—
Female	0.9 (0.5–1.5)
Education	
Under college	—
College and higher	0.8 (0.5–1.5)
Occupation	
Unemployed	—
Employed	0.9 (0.4–2.1)
Students, retired	1.6 (0.6–4.4)
Insurance status	
No	—
Yes	3.5 (1.9–6.2)***

^a^ Odd ratio.

^b^ 95% confident interval.

***p < 0.01.

## Discussion

The overall outpatient satisfaction of this polyclinic was not high (53.5%) and the five factors (facilities, services provision results, information transparency and administrative procedures, accessibility, and interaction and communication of staff) had a high internal consistency reliability, with a Cronbach’s Alpha of over 0.9.

The outpatient satisfaction (53.3%) was low when compared to previous studies on inpatients which ranged from 60% to more than 90% [1, 15, 20]. This low rate may be explained by the short time using healthcare services and the day-time treatment service. Patients may expect the same quality as hospitals offer, thus they may rate their satisfaction lower in the self-reported questionnaire.

Netemeyer et al. proposed four steps of the scaling procedure, including [1] construct definition, [2] generating and judging items, [3] designing and conducting studies to develop a Scale, and [4] Administration and Analysis [21]. In Vietnam, the Ministry of Health issued a criterion for measuring patient satisfaction, which was just implemented in step two by judging items by content validity and face validity. This study was conducted to continue the scaling procedure, and to assess the reliability and validity of the tool. In terms of validation, the study used EFA to determine patient satisfaction factors. According to Hair et al, a sample size should be obtained with the highest possible cases-to-variables ratio, or at least to have at minimum five times as many observations as the number of variables to be analyzed and the more acceptable sample size would have a 10:1 ratio [22]. The scale has 39 items, the sample size of the study was quite small with 301 patients, and the cases-to-variables ratio is about 7:1, which meant the study just had enough cases to carry out factor analysis. By running Bartlett’s Test of Sphericity and KMO with meaningful values (*p* < 0.05, 0.5 < KMO = 0.95 < 1), it was consolidated that the conditions were qualified to run EFA.

To assess the reliability of the tool, Cronbach’s Alpha coefficient was applied. All items on the scale with coefficient value > 0.7 were considered good as a rule of thumb [22]. The study chose the cut-off point for the five factors as the point at which the Eigenvalues explained 78.8% of the total variance. This value was relevant because Eigenvalue was the most reliable way to establish a cut-off on the scale of 20–50 variables [21, 22]. To determine the variable’s role and contribution in determining the factor’s structure, the Varimax rotation method was used because this method could give a clearer separation of the factors [22]. For the sample size of 301, Hair et al suggested that factor loading needs to be over 0.35 for significance [22]. The loading factors of all items in this study were above 0.5, which were considered practically significant.

The results showed that 39 items belonging to five factors with highly internal consistent reliability (Cronbach’s Alpha > 0.9). It means that this 39-item scale could be used to analyze patient satisfaction and its associated factors. Additionally, the scale in this study was a formative measure, although it had limitations because it was only suitable in a specific context and difficult to apply to other contexts, this kind of measurement has also been used in a number of other similar studies [23, 24]. The component structures in this study’s scale were considered as indicators for the points that patients were not satisfied with and need to improve, thereby orienting managers to act more accurately.

The study showed that among the five components evaluating patient satisfaction, patients were most satisfied with the factor of “Facilities” (mean score of 46.1). This result was different compared to other studies in which “facilities of the clinics” had low satisfaction [25, 26]. This could be explained by the fact that facilities in the clinic were built with new and clean equipment. Meanwhile, among the five components, the “service provision result” component had the second highest satisfaction score, namely 44.3. This result was not similar to some studies in Vietnam [10, 11, 27, 28]. This was explained by the fact that specialized physicians were recruited in the clinic, affecting the quality of examination and treatment results. In addition, the patients gave a lower score of satisfaction with the price information of the services and the explanation of the test results as shown in Table 2. This may be explained by the fact that the clinic had just operated with uncompleted procedures, which also led to a longer waiting times for patients, affecting patient satisfaction as some studies had mentioned [25, 28, 29].

In our study, we also analyzed the association between several independent variables with patient satisfaction. The result showed that only insurance status had an association with patient satisfaction. This result was similar to the results of some other studies conducted in Vietnam [10, 28]. It could be explained by the fact that in Vietnam, the uninsured payment process was more complicated than the insured payment process. Besides that, there was a belief that if using health insurance, patients would get health care of a lower quality than uninsured patients. Most people not using health insurance were in better economic conditions with higher expectations and requirements than insured patients. So, their satisfaction level was always lower than the satisfaction level of the insured patient. The group of patients not using health insurance needs to be examined further, especially in Vietnam where the rate of out-of-pocket health expenditure was close to 50% [30].

### Limitations of the study

This study has a few limitations. First, the primary data was collected from the outpatient in the clinic. A future study should be conducted with inpatients when the clinic is upgraded to a hospital. Second, this study is limited to a clinic where the results of patient satisfaction cannot be generalized to other clinics. A future study should be conducted in other clinics that represent the seven ecological regions of Vietnam.

### Conclusion

The satisfaction level of outpatients admitted to the polyclinic in Hanoi was low although the attitude and communication of health workers were good. Furthermore, the service provision results and the question of how to meet the needs of uninsured patients need to be considered in the future to improve the quality of medical examination and treatment. The modified tool for patient satisfaction assessment has high convergent validity and internal consistency reliability. Vietnamese and international health managers could use this modified tool to assess patient satisfaction, thereby finding solutions to improve health quality in healthcare facilities.
